# The Obscure Potential of AHNAK2

**DOI:** 10.3390/cancers14030528

**Published:** 2022-01-21

**Authors:** Mohamed Zardab, Konstantinos Stasinos, Richard P. Grose, Hemant M. Kocher

**Affiliations:** Barts Cancer Institute, Queen Mary University, London EC1M 6BQ, UK; m.zardab@nhs.net (M.Z.); k.stasinos@qmul.ac.uk (K.S.); r.p.grose@qmul.ac.uk (R.P.G.)

**Keywords:** AHNAK2, cancer, biomarker, screening, AHNAK

## Abstract

**Simple Summary:**

AHNAK2 is a relatively newly discovered protein. It can interact with many other proteins. This protein is increased in cells of variety of different cancers. AHNAK2 may play a vital role in cancer formation. AHNAK2 may have a role in early detection of cancer. This obscure potential of AHNAK2 is being studied.

**Abstract:**

AHNAK2 is a protein discovered in 2004, with a strong association with oncogenesis in various epithelial cancers. It has a large 616 kDa tripartite structure and is thought to take part in the formation of large multi-protein complexes. High expression is found in clear cell renal carcinoma, pancreatic ductal adenocarcinoma, uveal melanoma, and lung adenocarcinoma, with a relation to poor prognosis. Little work has been done in exploring the function and relation AHNAK2 has with cancer, with early studies showing promising potential as a future biomarker and therapeutic target.

## 1. Introduction

AHNAK nucleoprotein 2 (AHNAK2), initially called C14orf78, was discovered in 2004 while exploring the function of its sister protein AHNAK nucleoprotein (AHNAK), by knocking out the *Ahnak* gene in mice through homologous recombination [1]. It is a large protein, whose canonical sequence is approximately 616 kDa, comprising 5795 amino acids. Human AHNAK2 is found on chromosome 14q32 with a 15 kb open reading frame (ORF) [1]. Two further isoforms have been identified, isoforms 2 and 3, with masses of 85 kDa and 605 kDa, respectively [2,3].

AHNAK2, like AHNAK, is a tripartite protein predicted to be composed of 24 highly conserved repeat segments approximately 165 amino acids long, starting from a short non-repetitive N-terminal segment and ending with a C-terminal segment approximately 100-kDa in size [1,4]. It is predicted that the repeat structure consists of a basic framework of linked antiparallel β-strands with interconnecting peptide loops (characterised as β-turns) [1]. Although not proven experimentally, a self-optimised method of secondary structure prediction (Network Protein Sequence Analysis, France) predicts it to contain 12 β-strands per repeat sequence, with 28 antiparallel β-strands packed into a disk-shaped seven-bladed (propeller-like) structure [1]. The predicted structure, due to the similarity in length and distribution, is likely that of a propeller protein, such as those found in RCC1, G β-protein, and clathrin, which have been confirmed with x-ray crystallography [5,6,7]. These repeating sequences also include SH3 binding sites and translocation and assembly modules (TamB). The short N-terminus has a ~90–100 amino acid segment that functions as a PSD-95/Discs-large/ZO-1 (PDZ) domain. The long C-terminal region has a nuclear localisation signal [4,8].

As AHNAK2 shares many structural characteristics with AHNAK, and in light of AHNAK2-specific studies, we can hypothesise possible functions, locations, and interactions [4,8]. First, PDZ domains are one of the most common modular interaction domains, usually consisting of 80–90 amino acids arranged in six β-strands and two α-helices [9]. The PDZ domain at the N-terminus allows AHNAK2 to attach to other PDZ domains, lipids, internal peptide sequences, and C-termini of a variety of proteins [9,10]. Furthermore, the β-propeller structure they share would allow AHNAK2, like AHNAK, to interact with numerous ligands and proteins, thus forming a part of large multi-protein complexes and scaffolding networks [10,11,12].

AHNAK and AHNAK2 localise to the nucleus, cytoplasm, and plasma membrane of various cell types [8,10,13,14,15]. AHNAK2 can reportedly localise together with AHNAK to the sarcolemma, transverse T tubules, and Z-band regions of mouse cardiomyocytes [1]. There was colocalisation of anti-AHNAK antibody in both wild-type and AHNAK knockout mice with anti-RyR (Sarcoplasmic reticulum), anti-dihydropyridine (DHP) receptor (T-tubules and sarcolemma), and anti-α-actinin (Z-band regions) antibodies [1]. However, in contrast, a separate study of skeletal muscle showed no colocalisation of AHNAK or AHNAK2 with dihydropyridine receptors [14]. Through SDS/PAGE immunoblotting of sub-cellular fractions of mouse myocardium, the AHNAKs were found to be co-sedimented, mostly with the bulk of Z-band material with nuclei and myofibrillar aggregates and the DHP receptor (membrane vesicular fraction). Both AHNAK proteins are linked to the T-tubule membranes through interactions with the β2 subunit of cardiac L-type calcium channels [1,16]. This link suggests a role in the excitation and contraction coupling mechanism [1]. The role, function, and location of AHNAK, given its homology to AHNAK2, may help us understand the role of AHNAK2.

AHNAK, through its C-terminus (aa 5262–5643), interacts with β2 and β1a isoforms of native cardiac L-type Ca^2+^ channels (Ca 1.2, 1.1) [16]. In cardiomyocytes, it is phosphorylated by protein kinase A (PKA), which is activated by cyclic adenosine monophosphate (cAMP), which in turn is activated by β adrenoreceptors [13,16,17]. Normally, AHNAK limits the influx of Ca^2+^ into the cardiomyocyte. In the immune system, when phosphorylated, AHNAK allows an influx of Ca^2+^ resulting in an action potential [13]. However, upon AHNAK knockdown, T-cells and osteoblasts malfunction due to the lack of Ca^2+^ influx [17]. In fact, AHNAK-knockout mice were shown to be susceptible to *Bartonella henselae* infection because of CD4+ T cell inactivation [18]. AHNAK also attaches to both G-actin and cytoskeletal F-actin, which have an important role in the maintenance of calcium channels in cardiomyocytes, smooth muscle cells, and osteoblasts [11].

It has been proposed that AHNAKs may act as both a tumour suppressor and promoter [8,19,20]. Transforming growth factor-β (TGFβ) is known to mediate tumour metastasis through the activation of the epithelial to mesenchymal transition (EMT) [21,22]. AHNAK was found to promote TGFβ/SMAD3-induced EMT and cancer metastasis [23]. Actin-dependent pseudopodal protrusion and tumour cell migration, known determinants of EMT, were found to be reliant on four proteins; AHNAK, Septin-9, elF4E and S100A11 [24]. Furthermore, AHNAK was associated with migration and invasion in mesothelioma [25] and hepatocellular carcinoma [26]. *AHNAK* is part of a gene panel with inflammatory markers that predict poor survival in laryngeal carcinoma [27]. Interestingly, an abundance of AHNAK and annexin A2 were found in extracellular vesicles released by mammary cancer cells towards non-cancer mammary fibroblasts, indicating a role for AHNAK in vesicular communication promoting cancer progression [28]. In a different context, AHNAK may have a tumour suppressor role. In seven of eight triple negative breast cancer cell lines, AHNAK mRNA expression was downregulated, with expression being inversely correlated with tumour status, lymph node status, lymph node infiltration, TNM staging, and prognosis [29]; this tumour-suppressive effect was associated with both the AKT/MAPK and Wnt/β-catenin pathways [29].

The large structure of AHNAK allows it to function as part of large multi-protein scaffolding networks, particularly in cell-to-cell contact. Upon reaching confluency, AHNAK in canine kidney cells relocates from the cytoplasm to the plasma membrane, associating as a hetero-tetrameric complex with actin and the annexin2/S100A10 complex [8,30]. In a calcium dependent environment, cell-to-cell contact forms through the phosphorylation of AHNAK by AKT [30,31], which then changes the localisation of AHNAK from cytoplasm to plasma membrane, where it attaches to a complex with annexin2/S100A10 and actin, specifically at the adherens junctions [8,16,30]. This implicates AHNAK, and by extension AHNAK2, in cell-to-cell contact, membrane ruffling, endocytic events, and cytoskeletal scaffolding and structural reinforcement [30,32,33,34].

Initially, knockout of AHNAK in murine models created no phenotypic change, suggesting the possibility that AHNAK2 may be compensating for the loss of AHNAK in various functions [1]. It has now been noted that AHNAK-deficient mice have a leaner phenotype, with resistance to high-fat diet induced obesity. They exhibited impaired glucose tolerance and higher fasting glucose levels with decreased Akt phosphorylation and cellular glucose transporter (Glut4) levels [35]. A strong link between AHNAK and bone development and metabolism in mice was discovered, with a newer long-term study of AHNAK-deficient mice showing reduced growth of the skeleton with more fragile and fracture-prone bones. Micro-CT scans of these mice at various intervals confirmed shortened, weaker bones and a difference in morphology of facial bones [36].

Here, we present a comprehensive review of the current understanding of the structure, functions, locations, and biology of AHNAK2. The majority of work with AHNAK2 has been done in oncology, with some convincing evidence of a pro-oncogenic role, in contrast to the contradictory tumour suppressor and oncogenic roles of AHNAK [8,19,20].

## 2. Function

### 2.1. AHNAK2 in Normal Tissue

AHNAK2 has been proven to be a structurally integral part of costameres in skeletal muscles, together with AHNAK [8,14,14]. Mouse skeletal muscle fibres were stained for these two proteins, as well as α-actinin, myomesin and vinculin [14], leading to the conclusion that AHNAK2 colocalises with vinculin and α-actinin at the end of z-discs connecting costameres to sarcomeres in skeletal muscle [14]. AHNAK2 and AHNAK are both part of the costameric network, located between Z-disks and the sarcolemma, which functions as a mediator of lateral transmission of force from sarcomeres across the sarcolemma to the extracellular matrix [14,37]. A murine study looking at wound healing following femoral artery wire injury showed AHNAK expression in the endothelium, with AHNAK2 exclusively expressed in the cytoplasm of the neointimal and medial cells [38]. On knockdown of AHNAK, there was a delay in healing after injury with no change in expression of AHNAK2, providing evidence that both proteins were not redundant in naïve arteries, with a novel localisation of AHNAK2 in smooth muscle cells [38].

Although AHNAK has been localised to myelinating Schwann cells and is important for their adhesion to laminin, little is known about AHNAK2 in the nervous system [39]. Periaxin (PRX), with an important role in myelination, is abundant in the peripheral nervous system, and structural studies have shown homodimerization of AHNAK2 with PRX through domain-swapping of their PDZ-like domains [10]. PRX is most closely related to the AHNAK proteins, specifically AHNAK2, with a 57% sequence identity in the PDZ domain [10]. AHNAK2 shares in the PRX PDZ configuration, with six major β strands (instead of 6) and two α helices in one monomer, although due to the configuration of the β2 and β3 strands the peptide binding pocket is larger in AHNAK2 [10]. Corresponding strands form a 6-stranded anti-parallel β sheet between the two intertwined sheets of AHNAK2 and PRX PDZ domains [10]. Although not proven in vivo, experiments concluded that both their PDZ domains exhibited uniquely intertwined dimers with extensive three-dimensional domain-swapping [10]. This means that PRX and AHNAK2 are likely to add stability and order to large molecular complexes through this interaction. With this dimerisation, it can be hypothesised that, like AHNAK and PRX, AHNAK2 may have a myelination maintenance role and that mutations in AHNAK2 could disrupt this specific interaction with PRX [8,10,40]. Furthermore, PRX mutations result in various demyelinating peripheral neuropathies, such as Charcot-Marie-Tooth (CMT) disease and Dejerne-Sottas disease, again reflecting PRX’s major role in the myelination of peripheral nerves [10,41]. Whole exome sequencing of patients with autosomal recessive CMT in a Malaysian family showed gene mutation and significantly reduced expression of AHNAK2 at both the mRNA and protein level in fibroblasts [40]. We know that AHNAK and PRX are involved in large protein complexes with the plasma membrane, as well as the cell cytoskeleton; they are also involved in the packing of lens fibres and the development of the peripheral nervous system [8,39,42]. This novel link between PRX and AHNAK2 may open a window into a wide array of possible functions of AHNAK2, especially functions related to its ability to form stable multiprotein complexes through its PDZ domain. AHNAK2 may be involved in the development of the peripheral nervous system but this has yet to be proven in vivo.

### 2.2. AHNAK2 in Disease

Systemic Lupus Erythematosus (SLE) is an autoimmune disease affecting the skin, joints, kidneys, and other organs, with considerable genetic predisposition [43]. A large-scale exome-wide study of 5004 SLE patients and 8179 healthy controls in a Han Chinese population identified three novel coding variants and four new susceptibility regions for the disease [44]. AHNAK2 with *LCT*, *TPCN2* and *TNFRSF13B* encompassed three novel mis-sense variants and two non-coding variants with genome-wide significance (*p* < 0.001) [44]. This may present new insight into the biological mechanism of SLE; it was the first study to comment on a gene aberration of AHNAK2 [44]. Interestingly, AHNAK was found to be a novel auto-antigen in SLE [45]. A study looking at methylating substrates of SMYD2 found that the central repeating units of both AHNAK and AHNAK2 are mono-methylated by SMYD2 at multiple sites [46].

### 2.3. AHNAK2 in Cancer

AHNAK2 over-expression has been identified in various cancer cohorts, including pancreatic ductal adenocarcinoma, a largely incurable, aggressive, and silent malignancy [47]. A multi-gene biomarker panel (*TMPRSS4*, *AHNAK2*, *POSTN*, *ECT2*, and *SERPINB5*) for diagnosing PDAC has been proposed, by analysing various transcriptomic microarray repositories with micro-dissected and whole tissue samples (the Gene Expression Omnibus (GEO) database, the ArrayExpress repository, and the Stanford Microarray Database [48]). This study concluded that the 5-gene panel can attain 95% sensitivity and an 89% specificity in five separate validation sets for differentiating between PDAC (*n* = 137) and all of the normal tissues (*n* = 197), PDAC precursor lesions (*n* = 15), and chronic pancreatitis (*n* = 9) (Table 1) [48].

Two years later, a similar study created a 17-gene classifier from transcriptomic databases derived from patient pancreatic tissue, to differentiate between PDAC (*n* = 228) and PDAC precursor lesions (*n* = 28), chronic pancreatitis (*n* = 19), other cancers (*n* = 2630), and healthy controls (*n* = 40) from various sources and validated over seven datasets with considerably high sensitivity and specificity and an area under curve (AUC) over 0.95. This was further validated by RT-qPCR on biopsies from fresh-frozen cancerous (*n* = 9) and non-tumour (*n* = 2) pancreatic tissue, with the 17-gene panel being significantly upregulated in cancer tissue.

High or intermediated mRNA expression of this gene classifier was also found to be related to poor prognosis in a cohort of 178 patients from The Cancer Genome Atlas (TCGA) [49]. Furthermore, using immunohistochemistry, AHNAK2 expression was detected in 53% (*n* = 138) of human samples [49]. Although TFF1 and LAMC1 levels in patient plasma showed no statistical significance when checked by enzyme-linked immunosorbent assay (ELISA), there was no further validation of AHNAK2 in patient samples in this study or any others till now.

It is interesting to note that AHNAK2 with *SERPINB5*, *TMPRSS4*, and *POSTN* featured in the gene classifiers of both studies. AHNAK2 mRNA expression was found to be raised in seven standard pancreatic cancer cell lines (AsPC-1, BxPC-3, Capan-2, CFPAC-1, HPAF-II, PANC-1, and SW 1990) (*p* < 0.005) in comparison to an immortalised non-tumorigenic pancreatic epithelial cell line (HPDE-6) [50]. This further supports the hypothesis that AHNAK2 likely has an oncogenic role in pancreatic ductal adenocarcinoma.

A recent study looked at 17-gene expression microarray datasets from GEO, and AHNAK2 was found to be one of seven differentially expressed genes (DEGs) (*AHNAK2*, *CDH3*, *IFI27*, *ITGA2*, *LAMB3*, *SLC6A14*, and *TMPRSS4*) that differentiated between pancreatic cancer and non-tumour tissues with an AUC of more than 0.85. AHNAK2 was also significantly associated with poor prognosis [60]. AHNAK2 mRNA expression was also included in a 5-gene panel to train an artificial neural network to differentiate between PDAC and normal tissue with a resultant sensitivity of 87.6% and a specificity of 83.1% [51].

Looking at AHNAK2’s specific relation with PDAC, immunohistochemistry was performed once more, but with a larger cohort of patients. AHNAK2 was highly expressed in PDAC (*n* = 79) compared with adjacent normal tissues (*n* = 64) [61]. Furthermore, AHNAK2 overexpression was linked with poorer survival rates (*p* = 0.033) in PDAC patients, and both AHNAK2 expression (*p* = 0.003) and pathology grade (*p* < 0.001) were independent prognostic markers in Cox regression analysis of the cohort of patients [61]. AHNAK2 was found to be mostly localised in the cytoplasm and plasma membrane of the epithelial cancer cells [49,61]. So far, it seems that there is a strong link between AHNAK2 and PDAC, with evidence to suggest a link between AHNAK2 expression and tumour grade, prognosis, survival, and oncogenesis, with a strong potential to be part of a diagnostic or predictive biomarker.

In the search for a biomarker to differentiate between severe urocystitis with reactive urothelial atypia and carcinoma in situ (CIS) in bladder cancer, a novel approach of using Fourier transform infrared imaging (FTIR) for label-free tissue annotation with laser microdissection of the tissue section of interest and liquid chromatography-tandem mass spectrometry proteomic analysis was performed [55]. Out of 3515 identified proteins, AHNAK2 and Keratin 6A (KRT6A) were chosen for IHC validation, due to having the highest ratio of means (over 58 and 29 times more abundant in high-grade cancer compared with cystitis) [55]. AHNAK2 was found to be highly expressed in high-grade cancer tissue and CIS, with almost no expression in tissue with cystitis. This finding was verified in a large independent patient cohort of fresh frozen paraffin embedded (FFPE) tissue, including severe cystitis with reactive urothelial atypia (*n* = 108), low-grade bladder cancer (*n* = 84), CIS (*n* = 67), and invasive high-grade bladder cancer tissue (*n* = 67). This confirmed the findings, with AHNAK2 IHC staining differentiating between cystitis and CIS with a sensitivity of 97% and a specificity of 69% [55].

This result is not a huge surprise, as a 14-gene mRNA urinary panel that included AHNAK2 differentiated between control and bladder cancer tissues with a sensitivity of 89% and a specificity of 95% [62,63]. Although requiring further validation, this analysis provides a novel use of AHNAK2 as a biomarker for the detection of CIS reoccurrence or persistence after Bacillus Calmette-Guerin (BCG) treatment for CIS. It can also differentiate between low-grade and invasive high-grade bladder cancers with a sensitivity of 80% and a specificity of 86% [55]. A potential role for AHNAK2 exists as a urinary, tissue, and blood biomarker in bladder cancer.

Of all cancers, AHNAK2 has been most studied in depth in the context of clear cell renal cell carcinoma (ccRCC). ccRCC is the most common form of renal carcinoma, characterised by malignant epithelial cells with a clear cytoplasm originating from the proximal tubules [64]. Convincing evidence supports an oncogenic role for AHNAK2 in ccRCC [52]. Initially, it was selected for study through integrative data-mining of four datasets in the Oncomine database, comparing tumour transcriptomes with surrounding normal tissue [52]. AHNAK2 was one of the top 200 upregulated genes and one of the 45 genes uniformly upregulated in all studied datasets. AHNAK2 was found to be highly expressed in ccRCC clinical samples at the mRNA (*n* = 533, TCGA dataset) and protein levels (*n* = 355, institutional samples), in comparison to adjacent normal tissue. Furthermore, high expression in these datasets was associated with advanced stage, metastasis, and shorter survival [52]. When studying a large cohort of patient ccRCC samples (*n* = 355), immunohistochemical (IHC) staining localised AHNAK2 in the cytoplasm of cancer cells with a significant increase in expression in comparison to adjacent normal tissue; higher expression of AHNAK2 was associated with G3-G4 tumours (*p* = 0.013) and metastasis (*p* < 0.05) [52]. A Mantel-Cox test also found a correlation between IHC and shortened patient survival (*p* = 0.032). This primary in silico work provided strong evidence that AHNAK2 may have a link with tumour growth, oncogenesis, and disease progression in ccRCC, which is further elucidated through in vitro and in vivo experiments.

Lentiviral-mediated knockdown of AHNAK2 reduced cell proliferation, growth, and migration in the immortalised CAKI-1 (ccRCC) cell line (*p* < 0.01) and inhibited xenograft tumour growth in nude mice [52]. This is consistent with the in silico analysis, in that higher AHNAK2 expression was associated with a higher occurrence of metastasis. To further understand why that is the case, gene set enrichment analysis (GSEA) of high (*n* = 267) versus low (*n* = 266) AHNAK2 mRNA expression data from TCGA was undertaken, which reflected dysregulation of genes involved in fatty acid metabolism (*p* = 0.002) and the citrate cycle (*p* = 0.012) [52].

Expression levels of adenosine triphosphate citrate lyase (ACLY), acetyl-co-enzyme A carboxylase (ACC), and fatty acid synthase (FASN) in AHNAK2 knockdown ccRCC cell lines (CAKI-1 and 786-O) were reduced on both the mRNA and protein levels. Furthermore, Oil-Red-O staining was performed on these cell lines, with a smaller quantity of lipid droplets being produced in AHNAK2 knockdown cells. This analysis provides strong evidence linking AHNAK2 to the pathogenesis of ccRCC through the dysregulation of fatty acid metabolism and the citrate cycle, as both pathways are known to be dysregulated in cancer, providing energy maintenance and cellular nutrition, although the exact mechanism is not known [65,66].

**Figure 1:**
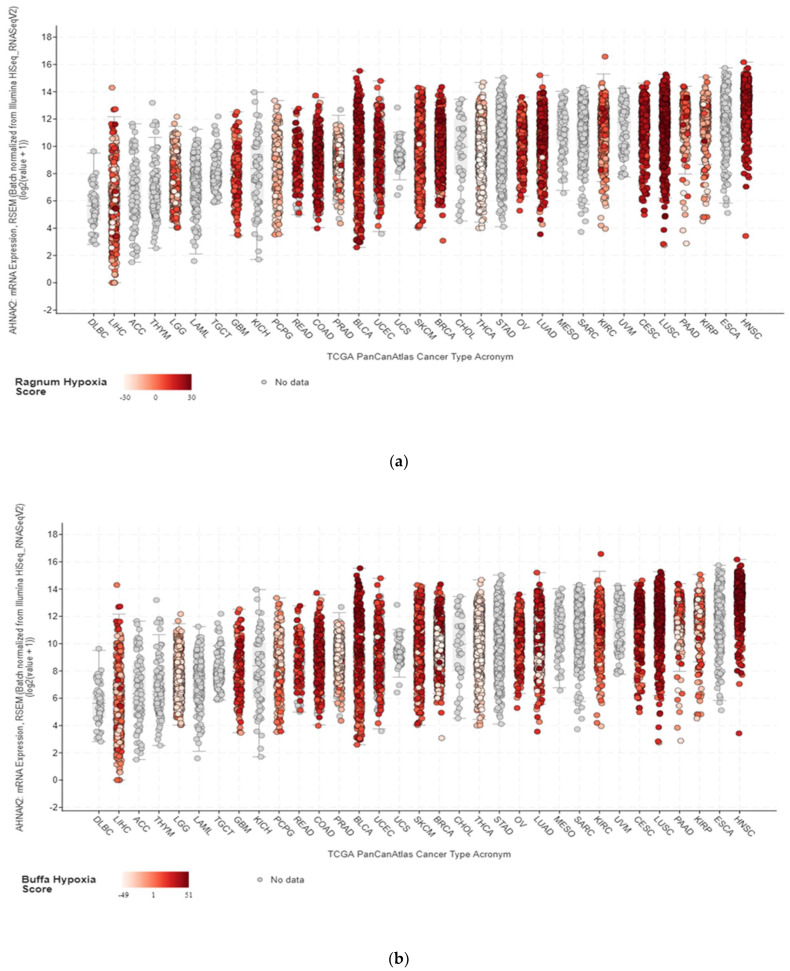
(**a**) Correlation of AHNAK2 mRNA expression with the Ragnum hypoxia score for the TCGA cohort of malignancies; (**b**) correlation of AHNAK2 mRNA expression with the Buffa hypoxia score for the TCGA cohort malignancies. (ACC: adrenocortical carcinoma; BLCA: bladder urothelial carcinoma; BRCA: breast invasive carcinoma; CESC: cervical squamous cell carcinoma and endocervical adenocarcinoma; CHOL: cholangiocarcinoma; COAD: colon adenocarcinoma; ESCA: esophageal carcinoma; GBM: glioblastoma multiforme; HNSC: head and neck squamous cell carcinoma; KIHC: kidney chromophobe; KIRC: kidney renal clear cell carcinoma; KIRP: kidney renal papillary cell carcinoma; LAML: acute myeloid leukemia; LGG: brain lower grade glioma; LIHC: liver hepatocellular carcinoma; LUAD: lung adenocarcinoma; LUSC: lung squamous cell carcinoma; DLBC: lymphoid neoplasm diffuse large B-cell lymphoma; MESO: mesothelioma; OV: ovarian serous cystadenocarcinoma; PAAD: pancreatic adenocarcinoma; PCPG: pheochromocytoma and paraganglioma; PRAD: prostate adenocarcinoma; READ: rectum adenocarcinoma; SARC: sarcoma; SKCM: skin cutaneous melanoma; STAD: stomach adenocarcinoma; TGCT: testicular germ cell tumours; THYM: thymoma; THCA: thyroid carcinoma; UCS: uterine carcinosarcoma; UCEC: uterine corpus endometrial carcinoma; UVM: uveal melanoma).

Hypoxia is one of the pathognomonic features of ccRCC, with the loss of von Hippel-Lindau tumour suppressor function (VHL) and dysregulation of hypoxia pathways [67]. For context, VHL, as an E3 ubiquitin ligase, was found to target the α-subunits of hypoxia-inducible transcription factors (HIF), specifically HIF-1α and HIF-2α [68,69,70]. HIF-1α activity has been found to reduce the tumour burden in ccRCC xenograft models, while HIF-2α promotes tumour growth [68,69]. Due to this, hypoxia-related pathways were studied and co-expression analysis of the Grumz Renal dataset provided evidence that there is a positive correlation between AHNAK2 expression and target genes of the hypoxia pathway (VEGFA, PDK1, DDIT4, and LDHA) [52]. cBioPortal mRNA analysis shows a strong correlation between high AHNAK2 expression and different cancers (Figure 1).

An in vivo study of various cancers, including prostate (DU145), lung (H460), breast (MCF-7), and renal (CAKI-1) cancer cell lines and normal (293T) cells, provided evidence that AHNAK2 expression increased gradually in a hypoxic growth environment (1% O_2_) in comparison to normoxia (21% O_2_). Knockdown of HIF-1α decreased the hypoxia-induced increase in AHNAK2 mRNA and the protein expression in CAKI-1 cells, while knockdown of HIF-2α had no effect (Table 2). HIF-1α interaction with AHNAK2 was further elucidated with chromatin immunoprecipitation assays on CAKI-1 cells showing hypoxia-promoted binding of HIF-1α to the AHNAK2 promoter region at hypoxia response elements 1 and 4 (HRE1 and HRE4) [52]. Furthermore, the growth of CAKI-1 cells in hypoxic environments significantly increased the protein expression of mesenchymal markers (n-cadherin, vimentin, and β-catenin), with decreased expression of epithelial markers (e-cadherin and ZO1) in comparison to normoxia. This hypoxia-induced epithelial-to-mesenchymal transition (EMT) of ccRCC cells did not occur during AHNAK2 knockdown. Therefore, AHNAK2 is essential for HIF-1α-mediated EMT in hypoxic conditions [52].

**Figure 2:**
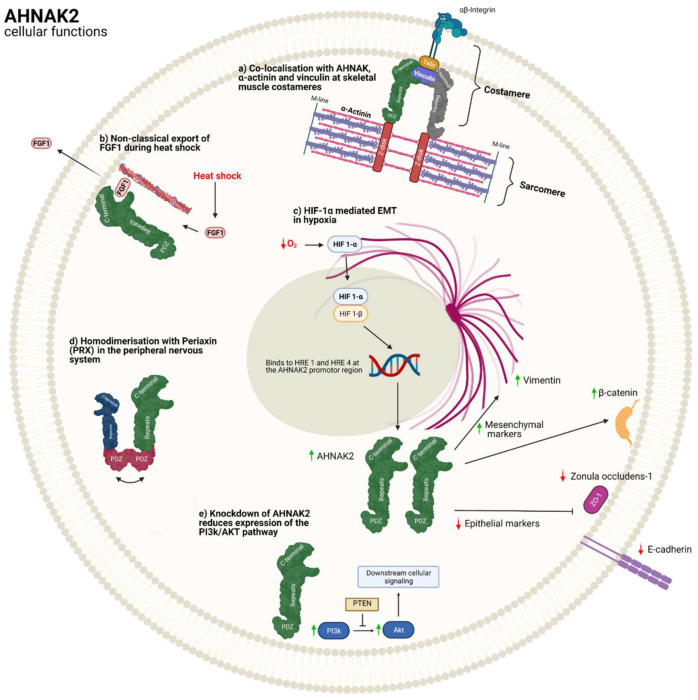
The main suggested cellular functions for AHNAK2.: (**a**) AHNAK2 was found to colocalise with vinculin and AHNAK at skeletal muscle costameres, linking the Z-disk to the costamere, which anchors the active unit of skeletal muscle, the sarcomere, to the sarcolemma (cell membrane); (**b**) FGF1 (fibroblast growth factor-1) is a non-classically released growth factor and signalling protein usually stimulated by stress, such as hypoxia and heat shock. AHNAK2s C-terminal was found to precipitate and translocate with FGF1 and F-actin at the cell membrane during heat-shock in murine cells; (**c**) clear cell renal carcinoma immortalised cell-lines (CAKI-1) have an HIF-1α- and AHNAK2-mediated epithelial to mesenchymal transition in hypoxic environments (1% O_2_); (**d**) X-ray diffraction of the PDZ domains of both periaxin and AHNAK2 provides a structural basis for the homodimerization of both homologues; and (**e**) knockdown of AHNAK2 in uveal melanoma immortalised cell-lines (M17, SP6.5) caused down-regulation of the PI3k/Akt/mTOR pathway, which is linked to controlling metabolism, proliferation, growth, and survival in cancer.

These findings are interesting, as we know that high expression of AHNAK2 is related to growth, proliferation, and metastasis, as well as hypoxia, yet this increase in expression in hypoxia is HIF-1α-dependent (Figure 2). Although HIF-1α has been described as a tumour suppressor gene in some papers [71,72] it is a known oncogene [68]. HIF-2α is more associated with oncogenesis in ccRCC [68]. The fact that AHNAK2 expression was HIF-1α-dependent rather than HIF-2α-dependent was explained by the link between HIF-1α and tumour metabolism (the Warburg effect), with glycolysis-related genes such as HK2, PGK1, CAIX, and LDHA being targets [52].EMT is also known to be a precursor to growth, invasion, and metastasis of cancers, particularly in the hypoxic environment of ccRCC [68,73,74]. Thus, with the GSEA data regarding lipid synthesis and the TCA cycle pointing towards AHNAK2 exerting an oncogenic effect in ccRCC through enhancing lipid synthesis, affecting tumour metabolism, and promoting hypoxia-induced EMT, these mechanisms may also explain the reduced proliferation of cells with shRNA knockdown of AHNAK2.

High AHNAK2 expression is linked to shortened patient survival and metastasis in uveal melanoma data (*n* = 63) from the Gene Expression Omnibus database (GEO) [53]. AHNAK2 knockdown in M17 and SP6.5 cells suppressed cell proliferation, migration, and invasion, while also inhibiting the activation of the phosphatidylinositol 3-kinase (PI3K) signalling pathway [53], which is known to be part of the PI3K-AKT-mTOR pathway that controls metabolism, proliferation, growth, and survival and is one of the most dysregulated pathways in cancer [75].

There is an increased expression of AHNAK2 in lung adenocarcinoma vs. pericancerous tissue [56]. Analysis of five Oncomine databases found increased AHNAK2 mRNA expression in lung cancer compared to normal tissue, and IHC slides from the Human Protein Atlas (HPA) database confirmed an increase in protein expression [57]. Clinicopathological grade correlation with AHNAK2 expression was performed on the TCGA and GEPIA database of lung adenocarcinoma patients, showing a significant positive correlation between high AHNAK2 expression and lymph node metastasis, staging, and poor survival [57]. Tissue microarray IHC of 235 lung adenocarcinoma patients confirmed the results of the in silico analysis [65].

Both RNA and protein expression were found to be increased in lung cancer cell lines A549 and H1299 compared to normal lung epithelial cells (BEAS-2B) [56]. In the presence of transforming growth factor beta 1 (TGF-β1), both cell lines had reduced invasion and migration in wound and Transwell™ chamber cell-based assays [56]. Western blot showed decreased SMAD3 phosphorylation in both lung cancer cell lines, with silenced AHNAK2 stimulated by TGF-β1, compared to normal controls. This could imply that AHNAK2 has an important role in TGF-β1/SMAD3 signalling [56].

GSEA and KEGG enrichment analysis of lung adenocarcinoma datasets showed a correlation between genes involved in focal adhesion [52], human papillomavirus infection, the PI3K-AKT signalling pathway [53], the regulation of actin cytoskeleton, and ECM receptor interactions with AHNAK2. Further comprehensive analysis of GSEA results revealed that AHNAK2 promoted tumour cell adhesion, cell–substrate junction formation, and regulation of the actin cytoskeleton and inhibited amino acid metabolism, cell mitochondrial respiration, and oxidative phosphorylation [57].

DNA methylation status analysis of cancer and normal cells from a patient with Epstein-Barr virus (EBV)-associated gastric cancer (GC) revealed increased AHNAK2 in cancer cells, with a possibility that silencing of AHNAK2 may increase chemosensitivity [54]. A study looking at alternative staging methods for gastric cancer performed Cox proportional hazard analysis on a set of enriched genes associated with disease to create risk score models of patients. AHNAK2 with *ABCC2*, *RNF43*, and *GSPT2* were chosen as a 4-gene signature highly related to disease progression in patients with unresectable metastatic GC, and this significantly increased the accuracy in predicting outcomes of GC in validation datasets (GSE-15081 and TCGA) [76]. Whole exome sequencing of ten thymic cancers (TC) from Japanese patients discovered focal genome copy number gains with elevated expression at the KIT and AHNAK2 gene loci, as well as other age-related mutations that are known to drive TC development [77]. Another exome study identified 323 somatic mutations from eight patients with pseudomyxoma peritonei, a rare malignant neoplasm of the peritoneum, and matched controls [78]. The most frequent were KRAS (G12D) (50%), ATXN1 (25%), and AHNAK2 (25%) [78].

There is another mechanism that may explain AHNAK2′s oncogenic effects and its participation in the stress-induced non-classical fibroblast growth factor 1 (FGF1) secretion pathway [79]. FGF1 is a non-classically released growth factor and signalling protein that is involved in various biological processes related to cell growth, tissue repair, morphogenesis, angiogenesis, and migration [80] Its release through direct translocation through the plasma membrane of cells is stimulated by different types of cell stress, namely hypoxia, heat shock, and growth factor starvation [80,81]. Through liquid chromatography–mass spectrometry and immunoprecipitation, murine (NIH 3T3) cells were cultured with adenovirally transduced FGF1 with a carboxy terminal HA tag. The team also tagged the C-terminal of AHNAK2 with V5 and co-transduced through adenoviruses’ expression vectors into the same cells [79]. AHNAK2 was found to be precipitated with FGF1 in the heat-shock-treated cells and colocalised at the periphery of the cell membranes [79]. AHNAK2 knockdown reduced FGF1 levels ten-fold when heat shocked but did not affect FGF2 levels or FGF1 levels in normally treated cells. We already know that AHNAK’s C-terminal binds with Annexin2/S100A10, PKC, F-actin, and α-actin at the submembrane of human cells [14,82,83]. Although this study took place with murine cells, it offered good insight at a novel function for AHNAK2, which may explain the oncogenic profile it has with ccRCC, PDAC, and UM [52,53,61]. It also provides more evidence of AHNAK2’s similarity to AHNAK in function and location with its colocalisation with F-actin, reflecting a shared link to the cell cytoskeleton [11,79,82].

Papillary thyroid cancer (PTC), with a metastasis rate of 5–10% [84], requires further research towards developing disease biomarkers. AHNAK2 is shows significantly higher mRNA expression in PTC tissue compared to adjacent normal tissue in the GEO, Oncomine, and GEPIA databases [58]. Kaplan-Meier survival analysis of the KM-plotter dataset showed poorer prognosis with reduced overall survival (OS) in patients with higher mRNA expression of AHNAK2 [58]. Analysis of tumour-immune cell infiltration using the tumour immune estimation resource (TIMER) and the CIBERSORT function showed a positive correlation between AHNAK2 expression and B-cell, CD4+ T cell, macrophage, neutrophil, and dendritic cell infiltration. No correlation with CD8+ T cells in PTC was found [58].

CXCL16 and AHNAK2 have been linked, both correlating with poor prognosis and elevated immune cell infiltration [85], with a previously demonstrated contribution of immune dysfunction to PTC progression [86]. This link forms a basis for further study of the role of AHNAK2 in PTC-immune oncogenesis. These data are further supported with the results of an ESTIMATE analysis showing a positive correlation between AHNAK2 expression and the proportion of immune and stromal cells in thyroid tissue [87].

Tumour-infiltrating cells are known to take part in the pathogenesis of lung adenocarcinoma [88], and TIMER analysis showed that high AHNAK2 expression was associated with more central memory CD4+ and CD8+ T cells, but reduced the expression of activated B cells, activated CD8+ T cells, eosinophils, immature B cells, DC cells, and all of their markers [65].

## 3. Conclusions

**Figure 3:**
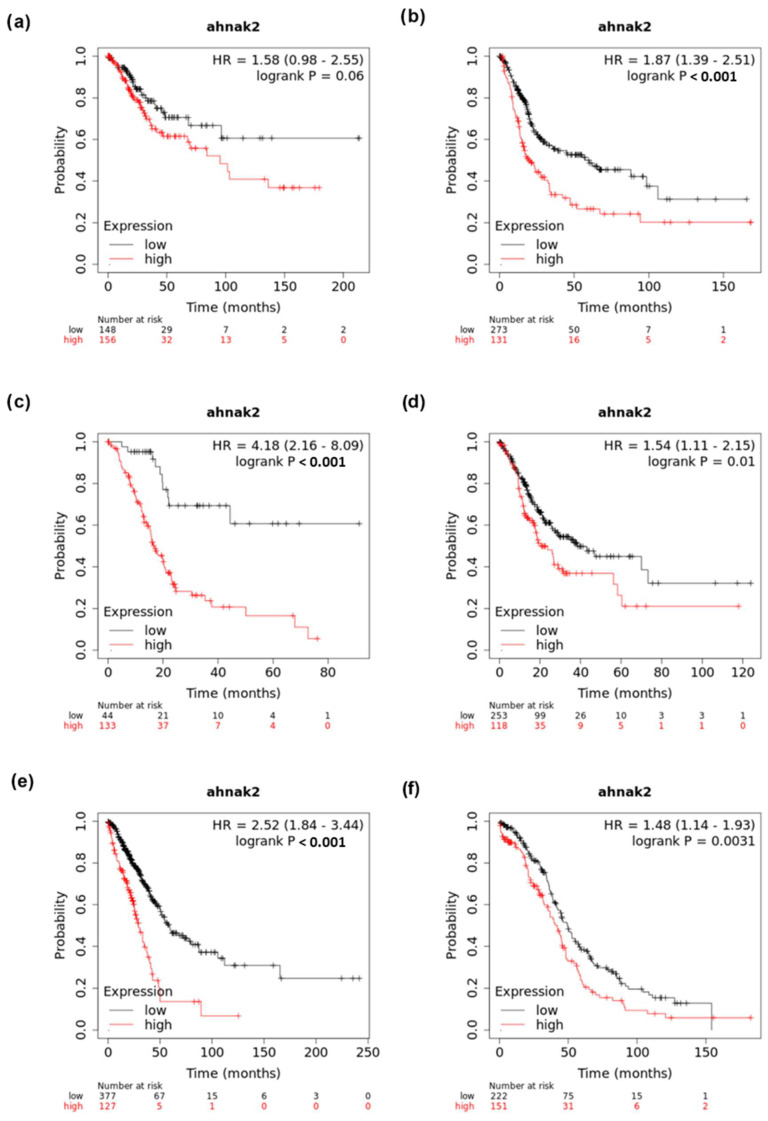
Kaplan-Meier plots generated via KMPlot (www.kmplot.com): (**a**) cervical squamous cell carcinoma, (**b**) bladder carcinoma, (**c**) pancreatic ductal adenocarcinoma (**d**) stomach adenocarcinoma, (**e**) lung adenocarcinoma, and (**f**) ovarian cancer.

AHNAK2 has a strong link with oncogenesis in pancreatic ductal adenocarcinoma, clear cell renal carcinoma, lung adenocarcinoma, gastric cancer, bladder cancer, and uveal melanoma (Table 1). Kaplan-Meier curves of AHNAK2 mRNA expression show worsening survival in multiple cancers (Figure 3). However, the biology of the protein is unclear, with early evidence linking HIF-1α mediated EMT in hypoxia and the PI3K/AKT/mTOR pathway with AHNAK2 as possible modes to increase tumour proliferation, migration, and survival. There seems to be a correlation between AHNAK2 and epithelial cancers that requires further investigation, particularly AHNAK2’s relationship with PDAC, as it is an aggressive tumour with limited treatment options. Apart from the molecular biology of AHNAK2 in disease, it would be important to see if AHNAK2 is detectable in patients’ bodily fluids, for possible use as a urinary biomarker in bladder cancer, including serum and plasma as possible predictive, diagnostic, or prognostic biomarkers.

A study looking at one case of EBV gastric cancer found methylation of AHNAK2, which is hypothesised to be the reason why EBV GC might have a high sensitivity to chemotherapy. AHNAK2 has a reported association with cisplatin (CIS) and 5-fluorouracil (5-FU) resistance, with 5-FU being used in colon, oesophageal, gastric, pancreatic, and breast cancers through the inhibition of thymidylate synthase (TS) and incorporating its metabolites into RNA and DNA. This may explain why AHNAK2 is associated with the progression of patients with unresectable metastatic gastric cancer and could provide an explanation for an AHNAK2-mRNA expressions link to worsening survival in PDAC, as advanced metastatic PDAC is now treated with FOLFIRNOX (Folinic acid, 5-FU, irinotecan and oxaliplatin).

Work must be done to delineate AHNAK2′s potential role as part of large multi-protein complexes attached to the cytoskeleton, and its cellular localisation within nucleus, cytoplasm. and plasma membrane in normal and pathological tissues. We do not have strong evidence for AHNAK2′s involvement in cell-to-cell contact, cytoskeletal stability, or cytoarchitecture, although structural analysis and similarity to AHNAK point towards that being highly likely. Further work on AHNAK2 interaction with known associated cytoskeletal proteins such as F-actin, α-actin, S100A10, Annexin2, and AHNAK will give us a better understanding of its basic functions. These basic functions may shed light on how HIF-1α-mediated hypoxia causes ccRCC to change phenotype from epithelial to mesenchymal cells. With high expression in epithelial cancer cells and a novel link to EMT, AHNAK2 may give us a better understanding of how the EMT process occurs, and could potentially provide us with novel targets for therapy.

There has been minimal work on the relationship between AHNAK2 and calcium channels, even though we know that the structurally similar AHNAK has its C-terminus attaching with L-type calcium channels to prevent cardiomyocytes or allow osteoblasts and CD8+ and CD4+ T cells’ influx of calcium into cells. Some TIMER analysis has been done in the context of LUAD and PTC, with AHNAK2 levels correlating with various sub-populations of immune cells, including CD8+ and CD4+ T cells, B cells, macrophages, and dendritic cells. AHNAK and AHNAK2 seem to colocalise in the sarcolemma of mouse cardiomyocytes, where voltage-gated calcium channels can be found. Therefore, there is good evidence for AHNAK2′s involvement in calcium homeostasis and, possibly, high AHNAK2 expression may cause dysregulation of the immune system through its effect on cytotoxic and helper T cells mediated via L-type voltage-gated calcium channels and calcium signalling.

AHNAK2 is needed for the non-classical secretion of FGF1 that can bind to fibroblast growth factor receptors (FGFR), exerting an effect on cell growth, tissue repair, tumour growth, invasion, and various cell survival related activities [81]. Further studies are needed to explore AHNAK2’s colocalisation with FGF1 in inflammation. Given that AHNAK knockdown caused impaired glucose tolerance and raised glucose levels in murine models, and as FGF1 has novel links with hyperglycaemic remission in mice [35], it is possible that other than the obvious cancer-related change of function, there may also be a novel link between diabetes and its management with AHNAK2, which may serve as an avenue of further research.

AHNAK2 in cancer has considerable promise for future studies, especially as it is not related to mutation but rather to the normal function of AHNAK2. Further understanding AHNAK2’s role in various cell types, its various isoforms, and its relationship with AHNAK, periaxin, and other cytoskeletal structures will be the gateway to understanding its overall role in disease.

## Figures and Tables

**Table 1:** A collection of all oncological studies of AHNAK2 with associated findings. (GC: gastric cancer; BC: bladder cancer; LUAD: lung adenocarcinoma; PTC: papillary thyroid cancer; TC: thyroid carcinoma; CPTAC: clinical proteomic tumour analysis consortium; and GTex: genotype-tissue expression).

Cancer	Type of Study	Experimental Environment	Findings	Reference
PDAC	mRNA microarray analysis	In silico analysis of microarray data from PDAC datasets	1. Part of a 5-gene panel that differentiated between PDAC, early precursor lesions, and non-malignant tissue.	Bhasin et al. 2016 [48]
PDAC	mRNA microarray analysis with in-vitro studies	In silico analysis of microarray data from PDAC datasets with qPCR and immunohistochemistry	1. Part of a 17-gene panel that discriminated between PDAC and non-tumour tissue in FFPE and fresh frozen tissue. 2. High protein expression in PDAC versus non-tumour tissue.	Klett et al. 2018 [49]
PDAC	mRNA microarray analysis	In silico analysis of microarray data from PDAC datasets	1. Part of a 7-gene panel differentiating between PDAC and normal tissue with a significant association with poor prognosis.	Almeida et al. 2020 [51]
ccRCC	mRNA microarray analysis with in-vivo and in-vitro studies	qPCR of ccRCC cell lines versus non-tumour cell lines with knockdown studies of EMT, hypoxia, and fatty acid synthesis	1. High expression in ccRCC samples versus non-tumour tissue. 2. Increased tumour proliferation, tumorigenesis, colony formation, and migration. 3.Upregulation in hypoxia with increased EMT and increased lipid droplets, compared to knockdown.	Wang et al. 2017 [52]
UM	mRNA microarray analysis with in-vitro studies	qPCR of UM cell lines versus non-tumour cell lines and knockdown studies	1. High expression associated with shorter overall survival time in UM with inhibition of the PI3K signalling pathway and increased proliferation, migration, and invasiveness of cell lines versus knockdown.	Li et al. 2019 [53]
GC	DNA methylation analysis	Immunohistochemistry and DNA methylation status of gastric cancer cell lines	1. Higher methylation in EBVGC cells compared to normal GC with a connection to 5-fluorouracil and cisplatin resistance.	Ohmura et al. 2019 [54]
BC	Proteomics study	Label-free Fourier transform infrared liquid chromatography-tandem mass spectrometry proteomic analysis of bladder cancer, urocystitis, and reactive urothelial atypia tissue.	1. Potential biomarker for bladder cancer, which can differentiate between urocystitis and low-grade carcinoma with invasive high-grade bladder carcinoma when tissue is stained for AHNAK2.	Witzke et al. 2019 [55]
LUAD	mRNA microarray analysis with in vitro studies	In silico analysis of microarray data for LUAD at TCGA, CPTAC, GEO and GTEx datasets of lung tissue samples with in vitro studies	1. AHNAK2 expression is upregulated in tumour samples. 2. Silencing AHNAK2 inhibits migration, invasion, and EMT in lung adenocarcinoma cells by repressing the TGF-β/Smad3 pathway.	Liu et al. 2020 [56]
LUAD	mRNA microarray analysis	In silico analysis of microarray data from lung adenocarcinoma datasets with tumour-immune estimation resource analysis.	1. Significantly overexpressed in lung adenocarcinoma and found to be an independent prognostic factor. 2. Negatively correlated to activated B cells and CD8+ T cells, while positively correlated to CD4+ T cells and tumour-associated macrophages.	Zheng et al. 2021 [57]
PTC	mRNA microarray analysis with in vitro studies	In silico analysis of microarray data from GEO, Oncomine, TCGA, and HPA datasets. IHC staining analysis and tumour immune estimation resource analysis.	1. Upregulation is significantly correlated to poor survival, advanced stage and grade. 2. Positive correlation with and B-cell, CD4+ T cell, macrophage, neutrophil, and dendritic cell infiltration.	Zheng et al. 2021 [58]
TC	mRNA microarray analysis with in vitro studies	In silico analysis of microarray data from TCGA. In-vitro studies of TC cell lines.	1. AHNAK2 is associated with a poor clinical outcome. 2. Inhibition of AHNAK2 suppresses the NF-κB pathway.	Ye et al. 2021 [59]

**Table 2:** Main signalling pathways affected by AHNAK2 in cancer. (ccRCC: clear cell renal carcinoma; UM: uveal melanoma; LUAD: lung adenocarcinoma; TC: thyroid carcinoma.)

Cancer	Signalling Pathway	Interaction with AHNAK2	Reference
ccRCC	(1) HIF-1 signalling pathway (2) Fatty acid and lipid synthesis	(1) AHNAK2 upregulated in hypoxia in a HIF-1α-dependent manner. (2) AHNAK2 upregulates ACLY, ACC, and FASN.	Wang et al. 2017 [52]
UM	(1) PI3k/AKT pathway	(1) AHNAK2 upregulates p-PI3k and p-AKT expression.	Li et al. 2019 [53]
LUAD	(1) TGF-β/Smad3 pathway	(1) AHNAK2 upregulates the TGF-β/Smad3 pathway through increasing the expression of p-Smad3.	Liu et al. 2020 [56]
TC	(1) NF-κB pathway	(1) AHNAK2 upregulates the translocation of the NF-κB p65 subunit into the nucleus and the phosphorylation of IKKβ, resulting in upregulation of the NF-κB pathway.	Ye et al. 2021 [59]
